# Autonomous Marine Robot Based on AI Recognition for Permanent Surveillance in Marine Protected Areas

**DOI:** 10.3390/s21082664

**Published:** 2021-04-10

**Authors:** J. Carlos Molina-Molina, Marouane Salhaoui, Antonio Guerrero-González, Mounir Arioua

**Affiliations:** 1 Department of Automation, Electrical Engineering and Electronic Technology, Universidad Politécnica de Cartagena, Plaza del Hospital 1, 30202 Cartagena, Spain; jcarlos.molina@upct.es (J.C.M.-M.); marouane.salhaoui@ieee.org (M.S.); 2 Laboratory of Information and Communication Technologies (LabTIC), National School of Applied Sciences, Abdelmalek Essaadi University, ENSA of Tangier, Route Ziaten, Tangier BP 1818, Morocco; m.arioua@uae.ac.ma

**Keywords:** ASVs, cloud computing, edge computing, artificial intelligence, object detection, marine environment monitoring, protected marine area surveillance, MPA

## Abstract

The world’s oceans are one of the most valuable sources of biodiversity and resources on the planet, although there are areas where the marine ecosystem is threatened by human activities. Marine protected areas (MPAs) are distinctive spaces protected by law due to their unique characteristics, such as being the habitat of endangered marine species. Even with this protection, there are still illegal activities such as poaching or anchoring that threaten the survival of different marine species. In this context, we propose an autonomous surface vehicle (ASV) model system for the surveillance of marine areas by detecting and recognizing vessels through artificial intelligence (AI)-based image recognition services, in search of those carrying out illegal activities. Cloud and edge AI computing technologies were used for computer vision. These technologies have proven to be accurate and reliable in detecting shapes and objects for which they have been trained. Azure edge and cloud vision services offer the best option in terms of accuracy for this task. Due to the lack of 4G and 5G coverage in offshore marine environments, it is necessary to use radio links with a coastal base station to ensure communications, which may result in a high response time due to the high latency involved. The analysis of on-board images may not be sufficiently accurate; therefore, we proposed a smart algorithm for autonomy optimization by selecting the proper AI technology according to the current scenario (SAAO) capable of selecting the best AI source for the current scenario in real time, according to the required recognition accuracy or low latency. The SAAO optimizes the execution, efficiency, risk reduction, and results of each stage of the surveillance mission, taking appropriate decisions by selecting either cloud or edge vision models without human intervention.

## 1. Introduction

There are unique areas in the marine environment that must be protected due to their singular characteristics and high environmental value. These habitats are particularly sensitive to alteration or disturbance by humans, changes in the ecosystem, or changes in climate. One of the legal tools for their protection is the declaration of the area as a marine protected area (MPA), which legally allows for the establishment of a scenario of maximum protection [1]. The main purpose of MPAs is to regenerate fishing resources, preserve natural resources, conserve marine species, and recover ecosystems.

A marine reserve is defined as a category of marine protected area with legal protection mainly against fishing or development. The main limits, generally, are that professional fishing (with the exception of a few authorized boats) and diving (also with authorized exceptions), recreational fishing, underwater fishing, and anchoring are totally prohibited. These activities in marine reserves must be monitored by the authorities to guarantee the care of the ecosystem by law [2]. A marine reserve can be made up of a single area or different non-adjacent areas and contains at least one integral reserve, which is a natural space with high ecological and biological value due to a unique and delicate ecosystem which is sensitive to any alterations.

The restrictions are even stricter in integral reserves: all activities are forbidden, with the exception of authorized scientific activities and sailing at a limited speed. In Spain, there are a total of eleven marine reserves [3]; four are on islets, islands, and reefs far from inhabited areas and ports, and the rest are on the coast or near inhabited areas. The surveillance of areas far from the coast is a real challenge: inspection vehicles must be autonomous and must not be compromised by the risk of becoming adrift. Long-distance communications with the land-based station must be fluid and stable, especially if 4G or 5G coverage is not available, as in most marine areas far from the coast, and in the video surveillance scenario, image transmission requires a highly stable bandwidth. All these restrictions are a major challenge for the surveillance of marine reserves on the high seas.

Several measures have been adopted for the monitoring and surveillance of marine reserves. The materials and human resources are available to carry out routine inspections or to set up devices to detect illegal fishing. In general, all marine reserves are equipped with vessels, georeferenced cameras, night vision binoculars, telescopes and remotely operated vehicles (ROVs), among others [3]. However, all these measures and means have the same disadvantage: the lack of a permanent presence. Despite the measures adopted, it is not possible to permanently monitor the nature reserve with these means, and the identification and arrest of offenders is practically incidental. This is why it is very difficult to obtain records of those who have accessed protected areas and to obtain real-time alerts to identify those responsible in the event of damage or alteration to the ecosystem. Unfortunately, even with the means and resources described above, illegal activities such as anchoring or poaching still take place.

Protecting remote marine areas with the currently available means is not enough for their full protection, especially in integral reserves. The challenges are quite demanding, and even more so in permanent surveillance. Autonomous surface vehicles (ASV) are ideal in this scenario for autonomous navigation, but there is also another issue. In order to monitor remote marine reserves, the capacity to detect and identify specific types of vessels is required. Detection and identification abilities by humans are difficult to equal in this scenario, and only visual recognition technologies based on artificial intelligence (AI) and the Internet of Things (IoT) can offer a detection capacity close to human capabilities.

There are also other issues: mainly water quality, pollution, and the effect on the ecosystem. In a previous work, we proposed an autonomous system consisting of an autonomous solar-powered marine robot with specialized sensing systems [4], designed to carry out long-term observation missions in shallow water, collecting georeferenced oceanic data to monitor and analyze physical–chemical water parameters.

We therefore consider permanent surveillance and inspection of marine reserves to be vital. For this, we introduce the concept of the “watchdog”; a watchdog roams around an area (for example, a fenced-in area around a house). As soon as an intruder is detected, the watchdog alerts the owner and deters the intruder from entering. If they enter the premises, the guard dog chases them out. This concept, applied to autonomous navigation by means of ASV craft together with the concepts of Industry 4.0 and applied to marine environments, provides a powerful proposal for the permanent surveillance of marine reserves.

This paper proposes and evaluates an autonomous marine vehicle based on artificial intelligence, designed to recognize and classify vessels considered as potential risks according to their type and activity. Its main goal is to track and follow them in real time to obtain information (identification and position, video recording, etc.) using automatic image recognition. When a vessel classified by the algorithms as a potential risk inside an integral reserve is detected and remains in the same position for a certain period, it could mean illegal activity. In the experiment, the proposed autonomous guard based on the ASV was tested in this scenario in order to recognize, follow, and identify vessels based on an autonomous navigation and AI image recognition.

The series of requirements for this include the following factors: it cannot alter the ecosystem, so its energy source must be totally renewable; its capacity to detect and recognize target vessels must be precise and reliable; it must have the ability to distinguish between different types; and most importantly, the detection capacity should not compromise the ASV’s autonomy.

There are radar-based detection autonomous underwater vehicles (AUVs) with fast and accurate detection systems, but this is not enough for precise recognition and identification [5]. In Chang et al. [6], synthetic aperture radar (SAR) images are used together with deep learning (DL) algorithms to detect and recognize ships by means of a powerful CPU and local graphics cards and low computational time, but these have a high power consumption, which is incompatible with a stand-alone vehicle. Due to the inevitable vibration and constant movement of the autonomous vehicle, it is advisable to use single board devices and CPUs. This type of solution is suitable for fixed surveillance stations, but not for autonomous vehicles, whose autonomy is compromised. On the other hand, fixed stations are not applicable in this case due to several factors, such as limited monitoring range, low reaction capacity, exposure to environmental conditions and marine environments (in the case of buoys), and ecosystem alteration (in case of installations on islets or reefs), among others. In Li et al. [7] a unified energy management framework was used to enable a sustainable edge computing paradigm powered by distributed renewable energy resources. Edge computing technologies significantly simplify local computing capacity and increase energy efficiency, while maintaining low latency. AI-based technologies such as edge and cloud computing have proven to be accurate in terms of recognition results and data analysis [8,9,10]. In Salhaoui et al. [11], hybrid use of cloud/edge technologies is considered optimal, significantly reducing the percentage of local computing by deriving most of the calculation to remote (cloud) servers and highly optimized algorithms through suitable and specific training processes.

As the paper’s main contribution and novelty, we propose hybrid cloud/edge technology, optimized for high image recognition accuracy, minimum power consumption, and low latency, in order to increase vehicle autonomy and efficiency, and increase the likelihood of mission success and security during autonomous surveillance missions in marine reserves with maritime autonomous surface ships (MASSs). High power consumption compromises vehicle autonomy during image recognition and identification, and high latency compromises the control and tracking algorithms. To select the most appropriate technology according to the scenario and circumstances, we propose the Smart Algorithm for Autonomy Optimization by selecting the proper AI technology according to the current scenario (SAAO). This algorithm is optimized to select the appropriate technology (cloud or edge computing) according to the situation and circumstances.

The rest of the paper is structured as follows. Section 2 outlines the current state of the art and related work. Section 3 describes the proposed platform for surveillance in marine reserves. Section 4 outlines the AI recognition and the proposed algorithm for autonomy optimization, while Section 5 describes a case study in the form of experimental tests.

## 2. Related Work

Unmanned surface vehicles (USVs) are the main investigation areas of maritime autonomous surface ships (MASSs), being used in surveillance, research, scientific investigation, security, etc. USVs are defined as self-contained unmanned, untethered vessels that can transit on the surface of the water autonomously or be remotely controlled [12].

USVs are currently the subject of a number of publications relating to disaster management [13]. They can play a vital role in disaster research [14] by replacing response teams in remote and hazardous areas [15], or by carrying out long-term monitoring [16]. Monitoring covers environmental areas such as water bodies [17], and ocean survey applications related to weather/storm prediction, water sampling and oil spill mapping/clean-up [18]. USV and AUV multi-vehicle collaborative navigation has also been tested in the underwater detection of hydrocarbons and oil spill surveys [19]. Through detailed maps and satellite navigation, an ASV can detect and avoid static obstacles; for example, Heidarsson et al. [20] proposed an approach using Google Maps to build a map of static obstacles. However, a new map is needed for dynamic obstacles that are continuously on the move. To respond quickly and effectively to the challenges of a highly dynamic environment, the ASV needs on-board logic to monitor the scene, identify critical situations, and perform appropriate route modifications [21]. An outstanding feature is its capacity to recognize an obstacle at a safe distance and avoid a collision by changing its course. Kristan et al. [21] proposed a new graphical model that supplies fast and continuous obstacle image-map estimation from a single video stream captured on-board a USV. In the field of unmanned ground vehicles (UGVs), the challenge of obstacle detection and distance estimation has been explicitly addressed, although in ASVs this is still a challenge; certain solutions applied to ASVs were inspired by the experience gained in UGVs. The obstacle sensors first used in UGVs and later in ASVs are radar (radio detection and ranging), sonar (sound navigation and ranging), stereo and monocular cameras, and LiDAR (light imaging, detection and ranging) [22]. LIDAR obtains excellent results for UGVs [23], although they have a high cost and a limited range. In order to overcome the drawback of the range and cost of scanning LiDARs, Montemerlo et al. [24] and Dahlkamp et al. [25] tackle the issue of low proximity path detection with laser scanners by segmenting color with the laser output. Near path points are identified by the laser, projected to the camera, and used to learn a Gaussian blend model that in turn is used to segment the rest of the captured image by the camera. Sorbara et al. [26] developed an embedded sensor that consists of a single-beam long-range laser range finder (LRF) mounted on a powered platform along with red–green–blue (RGB) and thermal cameras. The cameras are employed to identify potential obstacles, with the distances determined by the LRF.

Although RADAR’s range is excellent, its size makes it more suitable for larger vessels and it has a poor performance in detecting small targets nearby [27]. Despite the expense involved, ships are best detected and identified by human observers. With the emergence of graphics processing units (GPUs), deep learning methods for computer vision, human intervention can be replaced by automatic detection by deep learning methods and synthetic aperture radar (SAR) imagery [28]. Small ASVs are usually battery operated, so power consumption is an important factor with a strong influence on range. ASV cameras have the advantages of low power consumption and are cheap and lightweight compared to other range sensors. Neves et al. [29] implemented stereo vision features on an ASV using a single onboard computer; however, detection was restricted to pre-defined buoys and targets.

In order to ensure accurate detection and tracking of objects at sea, autonomous vessels require a range of sensing capabilities. Radar can provide such an overview, although certain small vessels and floating objects are challenging to recognize. Computer vision by onboard cameras can be used for this as a reasonable alternative to a human lookout [30]. The work proposed in Prasad et al. [31] examines the technical challenges of marine image processing and artificial vision problems for video streams generated by cameras. These challenges include the dynamic nature of the background, the lack of static cues, the presence of small faraway objects, and lighting effects. Wawrzyniak et al. [32] propose a method of identifying and tracking vessels using video streams of existing port and river surveillance systems. The method detects all types of moving vessels, operates under varying lighting conditions, and assigns a unique identifier to each vessel detected.

Ferreira et al. [33] proposed a system that aims to provide fishing management and monitoring systems including the identification of the vessel registration number, and the control of the entry and exit of fishing vessels in and out of port. The data captured by the cameras were evaluated using a computer vision approach to identify the vessel’s registration number, and a historical log of entries/exits from the port is created. In Cho et al. [34], using a monocular camera mounted on a USV, automatic feature extraction and tracking filter algorithms are applied for real-time vision-based detection and tracking. The approach aims to detect and track another surface vessel by deploying computer vision techniques.

Novel technology has already been deployed on autonomous craft as part of the Marine 4.0 concept, where AI, cloud, and edge technologies are of great importance. For instance, the IBM-funded project, the Mayflower Autonomous Ship (MAS), will use the IBM power servers, IBM Watson AI, cloud, and edge computing technologies to navigate autonomously and avoid ocean hazards as it travels from Plymouth (England, UK) to Plymouth (Massachusetts, USA) [35], thus expanding knowledge of the ocean and removing barriers to marine research. In [36], a Google Cloud Machine Learning Engine is used to deploy an AI-based object classification system: a software suite for detecting, identifying, and tracking surface objects. It makes ships safer and more efficient by automatically analyzing data from a number of new sensors, along with the ship’s own automatic identification system (AIS) and radar.

The heterogeneous computing capabilities available at the edge as a combination of microcontroller coprocessors, CPUs, programmable gate arrays (FPGAs), digital signal processors (DSPs) and AI acceleration devices can be energy efficient by allocating different workloads to the most efficient computing engine [37]. This architecture of different interconnected hardware elements is of interest in fixed stations where autonomy or power is not a constraint. Besides latency, edge computing is also able to address security and privacy requirements by preventing the transmission of sensitive or identifiable data over the network [38]. In Zhu et al. [39], edge computing is used for monitoring marine fishery vessels and improving the real-time performance of the system in the case of restricted marine communication, but no illegal fishing or activities detection are contemplated. Longépé et al. [40] attempts to monitor the amount of illegal fishing in the Arafura Sea, by detecting fishing vessels from a spaceborne vessel detection system (VDS) based on high-resolution radar imagery and the satellite-based automatic identification system (Sat-AIS) from shore stations. Detection is based on the verification of AIS messages to a shore station. Being useful for monitoring, it has significant limitations in real-time responses when covering such a large area or when performing AIS-based vessel identification.

Some scenarios are especially demanding when requirements such as low latency, high accuracy, high autonomy, and low energy have to be guaranteed in the same solution. In these cases, the collaboration between cloud and edge computing platforms becomes of great interest. Ananthanarayanan et al. [41] describes a traffic density analysis in urban areas based on video identification performed on edge and cloud computing. They offload most tasks to powerful hardware on base stations in the edge side. In this scenario, energy is not a limited resource, and the large number of videos analyzed on cloud servers may result in high-latency but better analysis results. Video processing is executed locally with low latency or with assistance from the remote cloud if necessary. Ren et al. [42] proposes a similar scenario, where a cloud–edge collaboration system is investigated in order to optimize latency, accuracy, and performance in mobile data traffic. In both scenarios, low latency and high accuracy must be guaranteed. The main difference is that in the second scenario, energy is a scarce resource, computing capacity on the edge side is limited, and cloud–edge collaboration becomes essential.

## 3. Proposed Platform for Surveillance in Marine Protected Areas

The BUSCAMOS-VIGIA ASV was developed by DAyRA (División de Automatización y Robótica Autónoma) group at the Technical University of Cartagena (UPCT). One of its achievements is described in González-Reolid et al. [4], where we gave the ASV the capability to perform long-term missions to acquire data from multiparameter probes in the Mar Menor (Murcia, Spain) on factors to decide the urgency in inspecting a specific area based on fuzzy logic.

**Figure 1:**
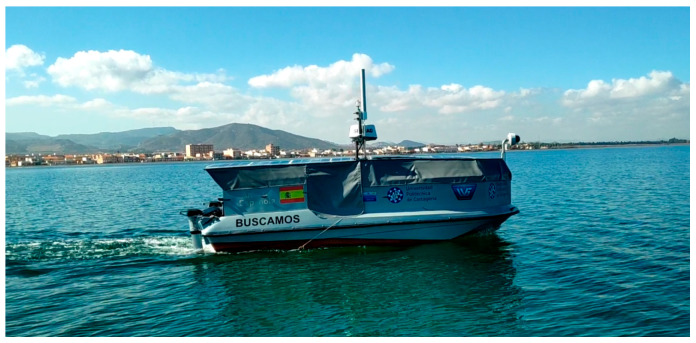
BUSCAMOS-VIGIA autonomous surface vehicle (ASV).

The most relevant characteristic of the ASV used in the experiment described in this paper is the vehicle’s size: it is 5 m long. It has a robust structure that protects the devices from the weather, as well as a sunroof. The inside of the vessel is subdivided into two sections by means of a bulkhead. In the stern are the elements related to power and propulsion: a block of eight 100 Ah batteries configured in two parallel lines, providing 48 V nominal power and 14 h autonomy. Two electric outboard motors, Torqeedo C4.0 Cruise model (Torqeedo GmbH, Gilching, Germany), allow it to sail at a maximum speed of six knots. It has two racks, located on the starboard and port sides. In the starboard rack are the IoT gateway (LattePanda single board computer, with 1.8 GHz Intel quad-core processor, 4 GB RAM and 64 GB on-board flash memory), and the Wi-Fi communications elements, energy management of different equipment, photovoltaic regulator, and electrical panel. The main elements of the port rack are the NI cRIO 9022 (National Instruments Compact Remote Input Output, Austin, TX, USA.) controller, the rudder controller, and the electronic periphery. It is equipped with side-scan sonar, echo sounder, GPS, inertial unit, and radar. It also has four LiDAR-Lite 3 (Garmin, Schaffhausen, Switzerland) devices in both bands, at the bow and stern, as safety elements for obstacle detection. It has a solar roof formed by five Enecom HF 130 panels that extend the autonomy of the vessel according to the environmental conditions, connected to a photovoltaic regulator and its battery pack. The battery pack can also be charged by AC chargers. In terms of vision, the ASV is equipped with an AXIS P5534-E PTZ camera (in the bow, Axis, Lund, Sweden) with 18× optical and 12× digital zoom (total 216×), with a resolution of 1280 × 720 p, as well as three additional Ubiquiti Air Cam cameras in the stern on both starboard and port sides. Its renewable energy source does not leave a carbon footprint, and it thus has no environmental impact, which makes it suitable for permanent navigation in marine reserves, particularly in integral reserves. Figure 1 shows a picture of the BUSCAMOS-VIGIA vehicle.

### 3.1. The ASV–IoT Architecture Development

**Figure 2:**
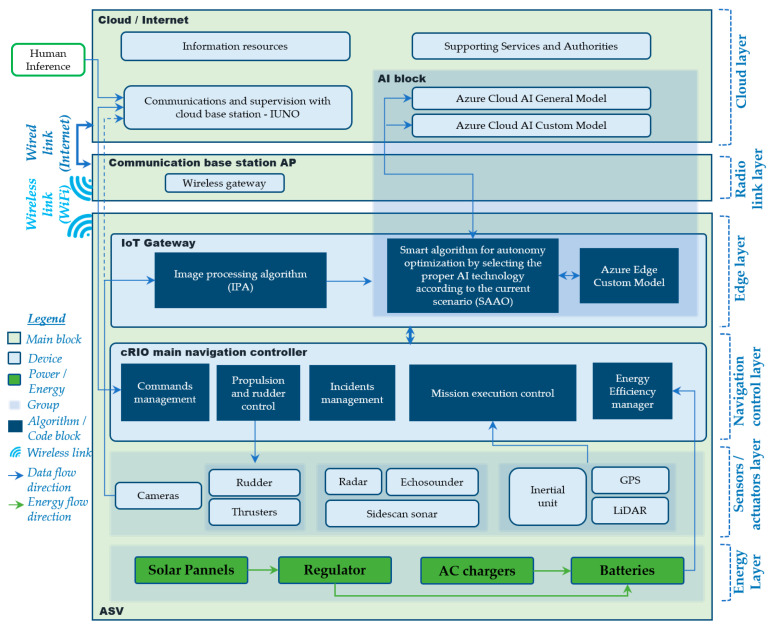
BUSCAMOS-VIGIA framework.

A framework describing the hardware and software architecture in the proposed system is shown in Figure 2.

The framework represents the whole system as follows: it is structured into two main blocks: the ASV block, communication base station access point (AP) block, and the cloud/internet block. The first represents the logic or physical elements included in the vehicle, and the second collects the elements in the remote station and cloud computing server. Each main block is classified into layers. The ASV block is divided into four layers: The energy layer, sensors/actuators layer, navigation control layer, and edge layer. The energy layer is formed of the elements that provide energy and autonomy to the vehicle. As can be seen, the batteries can be charged in two ways: through photovoltaic technology (during navigation—so as to extend the vehicle’s range—or in port) or through an AC source when solar power is not enough, or a quick charge is needed when moored. The next layer is the sensors/actuators layer, with different detection elements which provide information to the upper layers in the framework (such as GPS, inertial unit, LiDAR and cameras). Additionally located here are the rudder and thrusters. The upper layer is for navigation and control and consists of an NI cRIO controller (National Instrument Compact Remote Input Output) 9022 model and its peripheral elements and modules. The elements in this layer are responsible for autonomous navigation and use information from the sensors in the lower layer and the AI image recognition response obtained from the IoT gateway in the upper layer. The cRIO controller is formed of a main body, based on a processor and FPGA, together with a reconfigurable chassis, with a series of modules necessary for several communication protocols to command and acquire data from the lower layer, such as CAN-NMEA2000, I2C, RS232 and RS485, according to the current hardware architecture. There are also a series of code block and algorithms, described in Section 3.3. The upper or edge layer is formed by the IoT gateway, where on-board AI runs the algorithms in charge of the camera’s image processing and analyzing system.

The second main block, called “cloud/internet”, contains just one layer in the highest position of the framework, called the “cloud layer”, containing not only the AI services, but also information and resources provided in real time from authorities and services, which are essential for planning or modifying the ASV mission. Additionally, found here is the **I**nterface for **Un**manned Dr**o**nes (IUNO) software. The IUNO software platform was also designed by the DAyRA group of the Technical University of Cartagena. The platform manages the integrated control of multiple unmanned marine vehicles with the aim of simplifying maritime operations. The results obtained from each vehicle, regardless of its characteristics, facilitate the success of the operation with a high degree of automation. This software has already been used in previous experiments and operations, such as [4,11], and is the only point with human intervention. Activities such as mission planning or remote supervision are commanded and managed from here.

Between the ASV and cloud/internet blocks we find the communications base station AP block, containing the radio link layer. This provides high wideband, low latency, and long-range Wi-Fi communications between the vehicle and the land. It is formed by two Ubiquiti ROCKET M2 2.4 GHz modules (one on land, and another in the vehicle, UI, New York, NY, USA.) and its antennas, with Ubiquiti airMAX connection. Due to the characteristics of the communications scenario, the land antenna is the sector type and the on-board is omnidirectional. The land station is connected to the internet. This layer is especially crucial in areas where 4G and 5G cover is not available, as in most integral reserves.

There is an extra block in both the ASV and cloud/internet main blocks called the AI (artificial intelligence) block. As explained in Section 4, it is formed of the Azure cloud general model, Azure cloud custom model, and Azure edge custom model for AI recognition according to the smart algorithm criteria described in Section 4.4.

### 3.2. Implemented Vessel Recognition and Tracking Algorithm

In this section, we outline and itemize the development of the above-mentioned IoT–ASV autonomous system; specifically, the algorithm related to overall mission management and its stages, vessel recognition system, and the implemented tracking algorithm. It has five main blocks, namely, the IoT gateway, the IP cameras, the ASV control system, the remote-control station, and the cloud/edge AI image recognition source.

The overall mission is planned and triggered by IUNO software in the cloud base station by setting either the desired area of inspection or the desired waypoints. The navigation controller consists of four navigation modes: Main Mission Mode (MMM), where the vehicle navigates by following preprogramed tracks; Fixed Buoy Mode (FBM), where the vehicle stays at specific GPS coordinates while maintaining a fixed heading; Tracking Mode (TM), where the ASV follows a target (vessel) until specific conditions are met; and finally, Inspection Mode (IM), where once the target has been reached, the vehicle stays at a fixed distance and heading from it, in order to obtain and classify general information about it. Depending on the current navigation mode, there are a series of priorities, targets, and outputs, as shown in Table 1.

The navigation mode will change according to the scenario and the current stage of the general mission, as specified in Algorithm 1 and Figure 2. The IoT gateway connects the navigation controller and IP cameras with cloud services.

During the entire mission, the IoT gateway receives image data from the IP cameras and sensors (through the cRIO controller). If a vessel is detected, the results contain its classification (according to the trained AI models), a bounding box in the images (center, relative *x*–*y* position, and size) and accuracy (percentage). Likewise, the IoT gateway receives the image processing results from the AI recognition source for each photo sent. The AI source (edge or cloud computing) to analyze images is determined by the “Smart algorithm for autonomy optimization by selecting the proper AI technology according to the current scenario” (SAAO) described in Section 4.4. The AI source uses advanced learning techniques to analyze the results and send them to the IoT gateway. The results obtained from the AI source are used according to the specific target of each mission stage, as described in Table 1. This process is carried out throughout the mission.

Once the mission starts (MMM), the BUSCAMOS-VIGIA ASV follows the defined mission whilst analyzing images until a vessel is detected. The AI source then classifies it according to the trained AI models to determine the risk level. The mission mode then moves to the next stage, the Fixed Buoy Mode (FBM).

In FBM, if the vessel has been detected with a camera other than the bow camera, the vehicle will initially change its heading (stern: +180°, starboard: +90°, port: −90°) until the vessel is detected with the bow camera. IoT image processing is used with the navigation controller to perform heading modifications to keep the detected vessel in the center of the bow camera. Once the heading points to the target vehicle, the BUSCAMOS-VIGIA ASV will remain in that heading, regardless of the detected vessel’s behavior. The ASV can act as a fixed buoy or stay in same position and heading. This mode is used to study the target’s behavior by analyzing the bounding box (size and position) of the processed images for a specific period to determine whether the target vessel is immobile in the same position, which could mean a potential risk because it could be fishing or anchored in a protected area.

At this point, the mission changes to Tracking Mode (TM). The ASV starts navigating and tracking the target by using the bounding box’s analyzed image collection to fix and update the heading. The IoT gateway links up with the main controller to modify the heading according to the target’s position in the image, keeping it in the center of the bow camera’s field of vision. It will continue in this mode until LiDAR detects the target at a specific distance or if it leaves the protected area. If the TM is successful and the target is reached, several actions can take place, such as saving the vessel’s position or generating remote alerts.

When the target is reached, the last stage, Inspection Mode (IM) starts. The ASV will remain at a fixed distance and heading from the target vehicle as long as possible. The objective of this stage is to obtain information about the target and generate alerts in the remote base station.

**Figure 3:**
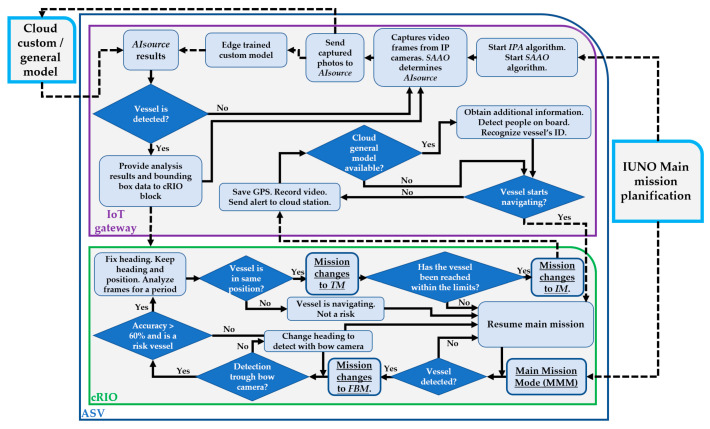
Platform’s communications in the tracking algorithm. IPA—Image processing algorithm.

This is described in Algorithm 1, as well as in the flowchart in Figure 3.
**Algorithm 1.** Vessel Recognition and Tracking Algorithm.1:**Start ()**2:**Step 1: While** (mission has not started) {}3:{Starts Main Mission Mode (*MMM*)}4:**Step 2: If** (mission has ended)5:{End ()}6:**Else**7:{Navigate follow defined mission}8:{Select the right AI image recognition source (*AIsource*) through *SAAO*}9:{Acquire frames from 4 cameras and send to *AIsource*}10:{Get the answer of every frame and add label with camera position (bow, stern, starboard, port) of detected vessel, called camera position (*CP*)}11:**If** ((accuracy of detected vessel > acceptable limits) **AND** (type of vessel == classified as potential risk))12:{**Go to** step 3}13:**Else**14:{**Go to** step 2}15:**Step 3:** 16:{Starts Fixed Buoy Mode (*FBM*)}17:{Get the answer of every frame and add label with camera position (bow, stern, starboard, port) of detected vessel, called camera position (*CP*)}18:**Switch** (*CP*)19:**Case** (*CP* == bow)20:**If** (accuracy < accuracy results acceptable)21:{Discard detected vessel. No risk.}22:{**Go to** step 2}23:**Else** 24:{Set new heading pointing detected vessel. Keep current position and heading}25:{Start study of target behavior by analyzing bounding box of images for a specific period}26:**If** (Detected vessel is in the same position)27:{Discard detected vessel. No risk}28:{**Go to** step 2}29:**Else**30:{Target vessel in the same position. Risk (anchoring, fishing)}31:{**Go to** step 4}32:**Case** (*CP* == stern)33:{Vehicle turns +180°}34:{**Go to** step 3}35:**Case** (*CP* == starboard)36:{Vehicle turns +90°}37:{**Go to** step 3}38:**Case** (*CP* == port)39:{Vehicle turns −90°}40:{**Go to** step 3}41:**Default**:42:{**Go to** step 2}43:**Step 4**:44:{Start Tracking Mode (*TM*)}45:{Navigate to track target vessel}46:{Acquire new frame from bow camera and send to *AIsource*}47:{Calculate the bounding box center and vessel position in order to fix heading while tracking}48:**If** (LiDAR detects vessel at 20 m)49:{Stop navigation. Target reached}50:{**Go to** step 5}51:**Else If** (vessel leaves integral reserve while tracking)52:{Target not reached}53:{**Go to** step 2}54:**Else**55:{**Go to** step 4}56:**Step 5**:57:{Starts Inspection Mode (*IM*)}58:{Acquire new frame from bow camera and send to *AIsource*}59:{Calculate the bounding box center and vessel position in order to fix heading and keep distance}60:**While** (Target vessel is stopped)61:{Record videos, save vessel’s position, obtain additional information, send data and alerts to cloud station}62:**If** (Vessel starts moving)63:{Stage finished. Information collected}64:{**Go to** Step 2}65:**Security Step**:66:**If** (Energy == 25%)67:{Return back to port area. Send alert to cloud station}68:**If** (Energy == 50%) 69:{Send alert to cloud station}70:**End** ()

### 3.3. ASV Control

As shown in Figure 2, our marine vehicle has a number of elements and devices interconnected through different networks. While the IoT gateway is in charge of image recognition and communications with the camera and the cloud, the cRIO controller is the ASV’s main control backbone. The National Instrument cRIO 9022 controller includes a real-time processor and reprogrammable FPGA through its LabVIEW environment [43], as well as a chassis that can be reconfigured according to the project architecture. By default, it comprises two Ethernet ports, a USB port, and serial connectivity. For this architecture, the chassis has been equipped with several modules that enable CAN-NMEA2000, I2C, RS232 and RS485 communications. Its specifications are a 533 MHz CPU, 256 MB DRAM, 2 GB storage, one Ethernet port, and other features listed in [44]. A consistent code for the cRIO controller was fully developed in the LabVIEW environment for ASV management, control, and command.

The software modules in the cRIO’s vehicle control program comprise these main operations, as shown in Figure 2:

- Commands management: It allows cRIO to dispatch commands from cloud station, such as receive and launch main mission after definition through IUNO software, stop it, or execute safety maneuvers;
- Propulsion and rudder control: Management of the different control loops for both propulsion and rudder, according to the obtained setpoint from mission execution control module;
- Incidents management: Security module that manages different actions depending on the incidents that may occur during the mission, such as loss of communications or the impossibility of continuing the defined trajectories due to external conditions, such as strong winds or rough seas;
- Mission execution control: This module manages navigation to each of the programmed waypoints, according to the running mission, by dispatching the different navigation commands for the heading and position control loops with the information received from sensors and IoT image analysis algorithm;
- Energy efficiency manager: The vehicle contains some non-critical navigation devices that can be disconnected in the event of energy and autonomy being compromised. This module executes the disconnection if required.

## 4. AI Recognition and Proposed Algorithm for Autonomy Optimization

### 4.1. Deep Learning for Object Detection

Rapid advances in DL and improvements in device capabilities, incorporating image sensor resolution, and optics, power consumption, memory capacity and computing power, have enhanced the cost-effectiveness and efficiency of accelerating the spread of vision-based applications. Compared to traditional CV techniques, the DL allows CV engineers to achieve greater task accuracy [38]. The neural networks used in DL are trained rather than programmed; therefore, applications using this method often require less expert analysis and tuning and leverage the large amount of video data already present in current systems.

Deep learning models can be called deep-structured neural networks. The history of neural networks can be traced back to the 1940s [45], and the original idea was to simulate the human brain’s system in order to solve general learning issues intelligently. A convolutional neural network (CNN) works by combining different layers of neurons that extract certain characteristics from the image. Each layer learns a different level of abstraction, and in the end gives a prediction of whether the object was detected or not [46]. Different online resources on deep CNN architectures and vision-related datasets have been implemented and are available on the internet. The potential of cloud-based platforms is expected to be exploited in the future for the development of computationally intensive CNN applications [10,47]. The obvious advantage is the possibility of creating intelligent systems with longer battery life, because the intense calculations are performed elsewhere.

Wide and deep CNNs present a major challenge for deployment and execution on resource-constrained devices. Cloud computing not only enables the handling of massive amounts of data, but also takes advantage of the benefit of high computing efficiency at a negligible cost. World leaders such as Google, Amazon, IBM, and Microsoft offer the public highly scalable, fast, and flexible cloud computing facilities to train CNN’s resource-hungry architectures. The cloud environment also facilitates setting up libraries for both researchers and new practitioners.

### 4.2. AI Cloud General Model Vision Solutions

Cloud computing is also impacting many applications that currently rely on local storage and processing power. Cloud computing provides computing resources in the form of a service or application over a network. Its services are generally divided into three categories: Platform as-a-Service (PaaS), Infrastructure-as-a-Service (IaaS) and Software-as-a-Service (SaaS). By remotely locating storage and processing capacity, image processing applications and machine vision systems can be performed remotely and paid for on a pay-per-demand or pay-per-use business model. Cloud-based systems optimally aim to automatically balance and distribute processing loads.

The computer’s field of vision is continually evolving; building a visual recognition model is a difficult and time-consuming task. In addition, the training of deep neural networks demand access to massive data and computing power, however this issue has also been overcome by new large-scale tagged datasets such as ImageNet [48]. Fortunately, there are many ready-to-run solutions on the market where these neural networks are often trained by lower-cost and more powerful clusters of cloud GPUs.

**Figure 4:**
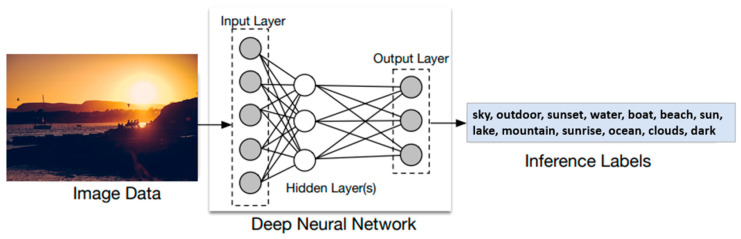
Object recognition of an image passing through a deep neural network (DNN).

These solutions were developed by several companies such as Google, Amazon, Microsoft, IBM, and others, and are provided in the form of application programming interfaces (APIs) which can be integrated into various application. Vision pre-trained models are either hosted for private use or offered as public services for deep learning in the cloud [49]. To use the pre-trained cloud-based models, application developers employ the cloud-exposed APIs to offload deep learning inference tasks to the hosting server (Figure 4).

**Figure 5:**
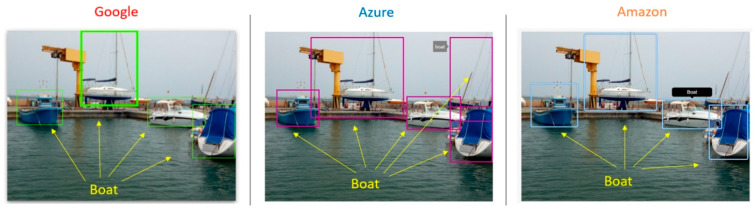
Comparison of three different clouds vision API detection of boats in Los Nietos port (Murcia, Spain).

Figure 5 shows an example of three different cloud vision APIs analyzing the same image, with different types of boats in a port. All three cloud services managed to detect most of the boats in the image. The bounding box location was accurate, although the cloud response could not exactly specify the type of each boat. Our objective is not only to detect each boat in the image but also to group them into more specific categories. The general model offered by the cloud has its limits in this regard.

### 4.3. Custom Model Training for Detection Specific Vessels

**Figure 6:**
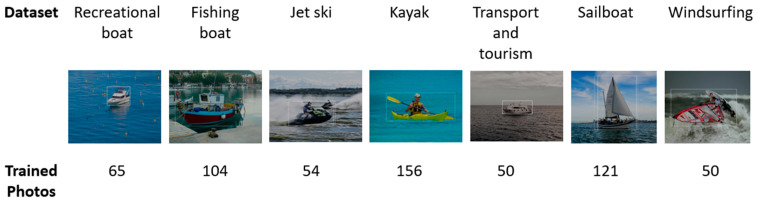
Types of vessels and number of images used to train the vision custom models.

The advantage of the customized model is the possibility of training it according to the use case, in addition to detecting the location of objects in the image. Our model has been trained to identify different types of vessels and their position in an image. We created our own custom object detection model to be implemented in the proposed IoT gateway using the Azure cloud service, because it supports the edge computing technologies and gives a better performance than the other solutions [11]. More than 600 photos of different types of vessels found around the inspection area in the experiment have been used to train the custom model. The model has been trained to recognize seven different types of vessels (Figure 6). The position of each vessel in the image was identified by drawing a bounding box around the object and providing the top and left pixel coordinates of the box, along with the width and height in pixels.

Microsoft Computer Vision reports that it uses a deep neural network called a residual neural network (ResNet) to train computers to recognize images [50]. The Azure Custom Vision machine learning algorithm trains on the submitted images and calculates its own accuracy by testing itself on these same images.

Azure can retrain our model in different ways, by quick training or advanced training by specifying the training computation time. The more time and pictures used to train the model in the platform, the better the results and performance will be.

**Figure 7:**
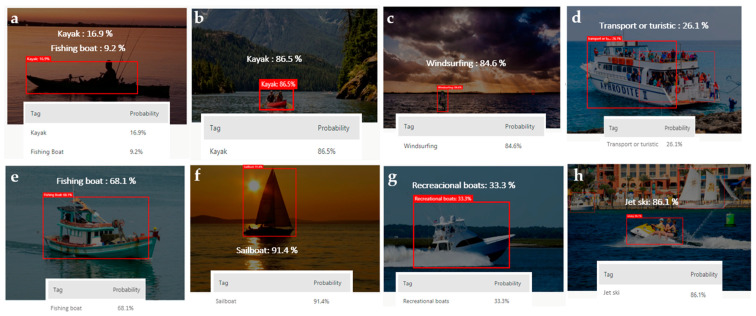
Performance of the cloud custom model object detection in discerning different boat types ((**a**): Kayak, (**b**): Kayak, (**c**): Windsurfing board, (**d**): Transport or turistic boat, (**e**): Fishing boat, (**f**): Sailboat, (**g**): Recreational boat, (**h**): Jet ski).

Figure 7 shows the detection testing of new photos not used in the training phase. The cloud trained model was able to differentiate between different types of boats; for instance, a man fishing in a kayak. The model detected the situation perfectly by the training photos.

Regarding meteorological conditions, in this use case there was no significant difference in accuracy results as long as the lighting was good enough. Cloud and edge custom models have a margin of adaptation to light conditions. In scenarios where light is insufficient, accuracy is affected. In some cases, even in low-light conditions, the model was able to detect the type of vessel with high accuracy, such as images 3 and 6 in Figure 7. It mostly depends on the pictures used in the training process.

#### 4.3.1. Cloud and Edge Custom Models

In the proposed architecture, we put forward the LattePanda IoT gateway running under Windows 10 LTSB OS, where the trained edge model has been deployed by using Microsoft Azure. Azure offers the possibility of choosing between different object detection custom model domains, namely, General, Logo, Products on shelves, and General Compact. The General domain is trained to be used only in the cloud (cloud custom model), while the General Compact domain is trained to be used in edge devices (edge custom model). The model performance varies by the selected domain; models generated by General Compact domains can be exported to run locally, so they are lightweight models and optimized for the constraints of real-time object detection on edge devices, although they are less accurate than the General domain.

**Figure 8:**
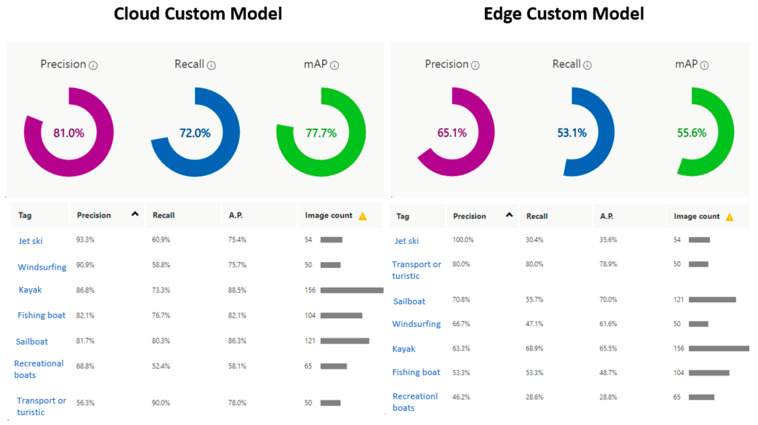
Performance differences between the edge and the cloud custom models

Figure 8 shows the training performance of 600 photos using the 7 h training budget. The edge and cloud models were trained with the same number of photos and training budget. The figure shows the difference between the two models after training.

**Figure 9:**
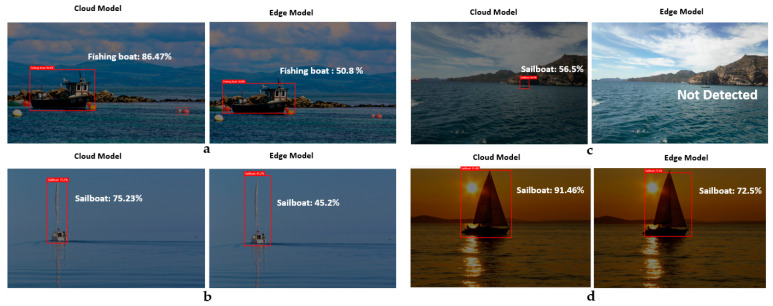
Cloud and edge custom models for detecting new vessels. ((**a**): Fishing boat, (**b**): Sailboat, (**c**): Sailboat, (**d**): Sailboat).

The models generated by the compact domains were optimized for the constraints of real-time classification on edge and mobile devices; therefore, they were slightly less accurate than a standard domain with the same amount of training data. Figure 9 clearly shows the difference between the custom model for cloud and edge uses, i.e., between the edge-trained model and the cloud-trained model. As can be seen, the distance from the object to the ships’ cameras affects the model’s percentage of accuracy. For instance, as shown in case 3 in the figure, the cloud model was able to recognize the vessel in the distance accurately, while the edge model was not.

#### 4.3.2. Latency Assessment in Edge and Cloud Custom Models

Performing powerful DNNs (deep neural networks) with real-time execution requirements on edge devices is still a challenge, regardless of the hardware acceleration and compression techniques deployed. Considering offloading the DNN computation from local devices to more powerful entities such as the cloud is a common scenario. Today, the cloud offers an edge model for deployment on tiny devices; however, cloud models are also needed to provide satisfactory performance. Another important factor to consider is that the cloud is known to facilitate storage, computational complexity, and the energy load on the edge and on local devices. Nevertheless, the cloud servers are topologically and spatially distant from the local stations, which causes significant communication latency. Real-time inference is absolutely required for many applications. For instance, frames from an autonomous vehicle’s camera must be processed rapidly to identify and evade obstacles, or a voice-based solution must have rapid analysis and understanding of the user’s input to provide a feedback. However, transferring data to the cloud for inference or training may result in more queues and delays in transmission from the network and cannot meet the stringent requirements of low end-to-end latency required for real-time interactive applications. For example, experiments have revealed that offloading a camera frame to Amazon Web Services and performing a computer vision task requires more than 200 ms of end-to-end data [51].

In this use case, Azure Custom Vision’s service works in Western Europe. The experiments were carried out in the IoT gateway mentioned above by using Python programming language. The results of the latency are summarized in Table 2, including average latency, standard deviation, and the minimum and maximum values calculated for each model.

The time that the cloud model needs to send the photos to the cloud for processing and receive the results back has been measured. Given that the trained edge model is migrated as a TensorFlow lite program, the photos are analyzed at the IoT gateway instead of being sent to the cloud. The performance of the edge models varies with the operating platform; hence, the inference time may vary. All samples were carefully and thoroughly checked for the same data on the same day. The experiment was repeated using the same data for both cloud and edge models. Each experimental campaign had about 300 different valid samples.

The results reported in Table 2 show the latency differences between edge and cloud models on the same machine. The average cloud model score is higher than the edge model by more than 1 s. However, the variance of the edge model is almost null compared to the cloud model, which is close to 900 ms, which justifies the edge model’s better stability in time than compared to the cloud model.

**Figure 10:**
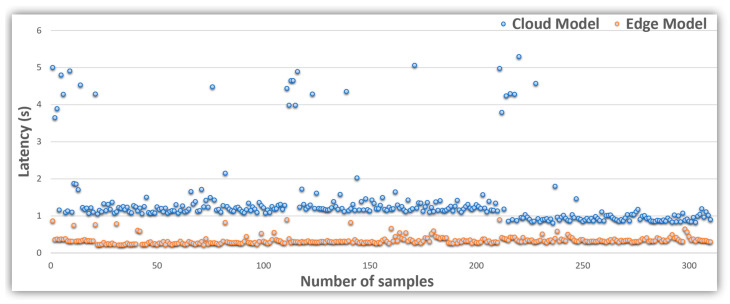
Latency of more than 300 samples.

Figure 10 compares the experimental latency results of both the edge and cloud models. The edge model showed more stability, and all values did not exceed the 1 s latency. The cloud model sometimes extended beyond 4 s. According to the cloud latency results, they can be classified into two ranges. The first extends for about 1 and 2 s, while the second extends for around 4 and 5 s. In addition, there is a band where no data have been registered, between approximately 2.5 and 3.5 s.

The cloud model’s apparent processing time instability can be explained by the internet connection volatility, because the photos have to be sent to the cloud model on remote servers for processing, unlike the edge model in which all photos are processed on board or at the local station.

The cloud and edge models were different in terms of accuracy, even though they were trained on the same reference images. In contrast to latency, the cloud model was more accurate, which eventually made it challenging to choose between both models. Real-time and high accuracy are both essential. However, in an autonomous marine vehicle where computing power is limited and environmental conditions are variable, low latency and high accuracy in every analysis are not guaranteed. The aim was to find an acceptable performance compromise, considering the evolution of the ongoing mission phases, as described in detail in the next section.

### 4.4. Smart Algorithm for Autonomy Optimization by Selecting the Proper AI Technology According to the Current Scenario (SAAO)

Maritime autonomous surface ships (MASSs) have to guarantee a series of requirements in order to fulfil their purpose; in particular, autonomy and security, while accuracy and latency in the image analysis are vital in the surveillance of marine reserves through AI-based visual recognition.

As defined previously, the surveillance mission is divided into four stages in order to optimize them according to the objectives and a series of priorities to attend to each stage, as defined in Table 1. Optimizing the mission execution means accomplishing it in the minimum time possible and with the highest guarantee of success, or in other words, execute every stage of the mission in the most efficient manner possible. An efficient mission means making the most of the energy available, a limited and essential resource for autonomous vehicles. The restrictive objective is to save energy and guarantee the success of the mission and its security by taking the appropriate decisions in real time, which is the crucial task of the proposed AI hybrid cloud/edge SAAO algorithm.

In stages where accuracy is a priority, optimizing mission execution means using AI to obtain the best recognition results, detecting vessels that are a potential risk to the marine reserve with the maximum precision and success. On the other hand, once the potential target has been detected and identified, optimizing the mission in stages in which latency is a priority means obtaining accurate results as fast as possible as a setpoint for the heading controller block.

**Figure 11:**
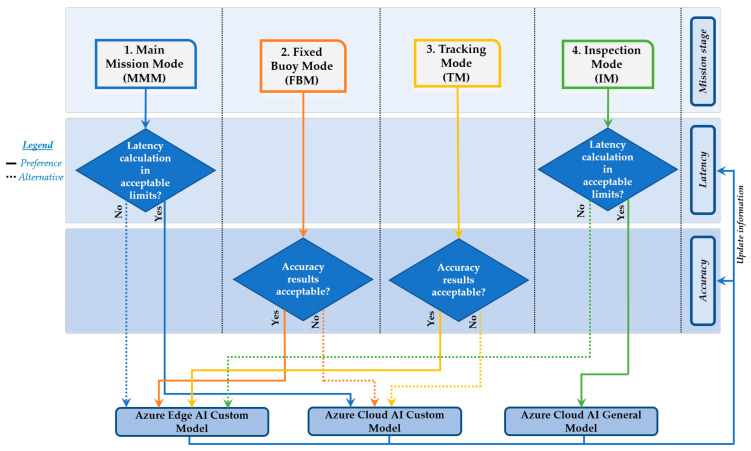
Smart algorithm for autonomy optimization by selecting the proper AI technology according to the current scenario (SAAO) diagram.

A single board low-power CPU is used to extend ASV autonomy; therefore, SAAO is designed to be efficient and executed in platforms where energy is a limitation. Using both cloud and edge computing technologies to analyze images at the same time will entail extra consumption and increased latency in the image analysis, especially in edge computing, where CPU resources are limited. Balance is the key to efficient and successful decision-making. These decisions are related to selecting the best AI source technology for the success of every stage, all of which have a series of priorities for optimizing the mission execution, as shown in Table 3:

In normal conditions, latency is adequate in edge models and accuracy is suitable in cloud models. This is the reason why there is a logic preference in every stage according to the defined priority, provided that accuracy is good enough. Knowing when and what to offload while maintaining real-time application quality of service (QoS) requirements are the challenges to overcome. Depending on the mission stage, accuracy, and latency results, a technique of offloading to edge computing device (IoT gateway) or remote cloud services is performed to complete its execution, as shown in Figure 11.

Basically, intelligent offloading can be used as an optimization-based approach with constraints such as bandwidth, network latency, accuracy requirements, or monetary cost. In this application, latency and accuracy have been defined as critical throughout the process, which is why the output results of the AI source are linked as inputs to the SAAO algorithm after analyzing the images.

The decision to offload or not depends on hardware capabilities, data size, the deep neural network (DNN) model to be used, and network quality, among other factors. These factors can be measured indirectly through latency and accuracy. Latency and accuracy are the main elements to be considered in this optimization approach. Figure 11 shows the SAAO diagram.

This diagram describes how the SAAO works. The latency and accuracy obtained from the previous analysis are analyzed according to the mission stage and the defined AI source preference. In the stages where latency is the priority, the accuracy result is analyzed to check whether it is within acceptable limits. This means that it should at least be able to identify the target and obtain its relative coordinates in the analyzed image in order to obtain the bounding box coordinates and use them as a setpoint for the heading control loop. There is no point in using a fast AI source if the algorithm cannot detect the target in its analysis. This is the critical line for accuracy.

In stages where accuracy is the priority, latency is analyzed in order to select the preferred or alternative AI source. The latency results may vary significantly depending on several factors, such as the percentage of bandwidth used, distance from ship to base station, or weather conditions, among others. With latency not being the priority in these stages, it has to be low enough to obtain an acceptably fast response, especially in the last stage, where the general cloud model is used to obtain general information about the target vessel. Latency is also crucial to keep the target in the center of the vision field.

Initially, latency values (average and standard deviation) are taken from Table 2 and Figure 10 results. Latency averages are updated for edge and cloud models with each new analysis. This determines acceptable limits for latency dynamically, considering parameters such as network quality and bandwidth indirectly.

(1) $$D_{BBC}=\;P_{n}-\;P_{n-1}$$

(2) $$T=\;T_{n}-\;T_{n-1}$$

(3) $$RS=\frac{D_{BBC}}{T}$$

**Figure 12:**
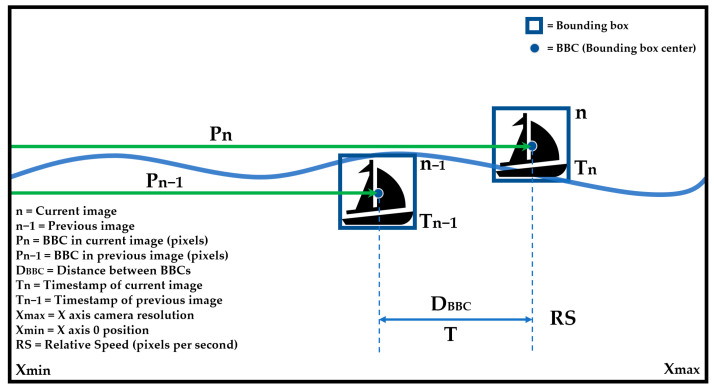
Calculation of acceptable latency limits. Main ASV camera point of view.

Figure 12 shows the difference of position between two consecutively analyzed images. 

From each successfully analyzed image, the bounding box of the detected vessel, its relative coordinates in the image, as well as its timestamp are obtained. By knowing the distance between bounding box centers (BBCs) and the time difference between analyses (T), the relative speed (RS) at which the target moves in the image can be obtained. During the time lapse between the analysis of two consecutive images, we approximate the relative speed of displacement of the target vessel and the ASV as a constant value, due to the considerable inertia of vessels at sea. With this information, it is calculated when the target BBC will leave the range of vision, and the SAAO can determine the selection of the preferred or alternative source of AI with regard to latency.

Several factors affect SAAO decisions, and they can be measured indirectly through latency and accuracy. Low latency and high accuracy are always desirable results, but every AI cloud or edge platform has its advantages and handicaps, and we cannot always achieve both simultaneously. Balance and effective decision making are the keys to make a mission efficient and successful.

## 5. Experimental Test and Results

### 5.1. SAAO Test

In order to test the decision making of the SAAO algorithm, an experiment was carried out based on the analysis of a 1.5 h video filmed in the Bay of Cartagena. The most interesting results were from a 2 min sequence of a fishing boat, whose results were analyzed as described below. A 10 s extract of the analysis is shown in this experiment.

**Figure 13:**
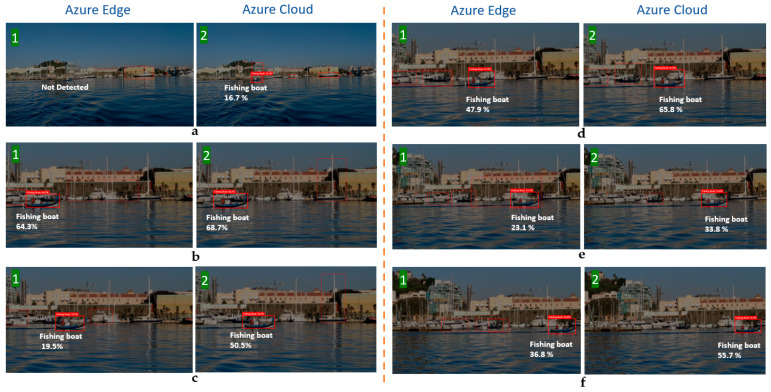
Cloud/edge results comparison of a fishing boat in different positions.

For the experiment, this video was used as the image source for the IoT gateway device, replacing the “Cameras” block shown in Figure 2. The captures extracted by the IPA (image processing algorithm) were analyzed in three different scenarios. First, only an edge computing analysis was carried out, recording latency and accuracy results, without the intervention of SAAO. The experiment was then repeated with the same images analyzed using cloud computing. Finally, the SAAO was tested in making decisions on the most suitable AI source for the analysis of the next image, based on the results obtained, and for each of the four stages, as shown in Table 4 and Figure 13.

As can be seen in Figure 13, there is a difference between the edge and cloud models when detecting the same image. Sometimes, the difference between the two percentages is not so significant, although in other cases there is a notable difference, especially when the boat is at a distance, which sometimes complicates the detection by using the edge model, as seen in the example of Figure 13a, or the latency results were high, in cloud computing mostly. These values condition the SAAO response, with different decision making in each stage, according to the preferred or alternative AI source. Special attention is paid to Figure 13a,e, where the low accuracy in the edge model and the high latency in the cloud model conditioned SAAO’s decision for the alternative AI model. In Figure 13f, the alternative AI model (according to Table 3) has also been selected in IM (Stage 4), due to the risk of losing the bounding box’s center of the target in the range of vision.

### 5.2. Experiment of BUSCAMOS-VIGIA with SAAO

**Figure 14:**
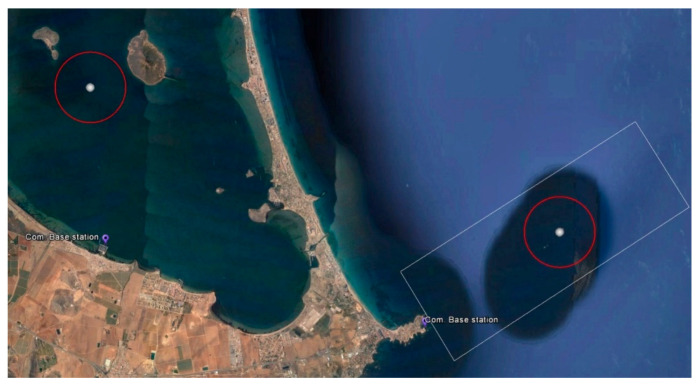
Scale experiment. Equivalence of area and distance from integral reserve (Islas Hormigas) to base station (**right**) and equivalent area in Mar Menor (**left**).

As mentioned in the Introduction, the complexity of autonomous surveillance varies significantly in different scenarios in the Spanish Network of Marine Reserves. The Cabo de Palos and Islas Hormigas Marine Reserve [52] in the Region of Murcia (Figure 14) has medium complexity according to the previously defined criteria. This reserve covers an area of 18.98 km^2^. This marine reserve is very near the Mar Menor, the largest saltwater lagoon in Europe, which was selected as the scenario for the case study because the water there is usually calm and winds are light, more suitable to BUSCAMOS-VIGIA prototype vessel (Figure 14).

The test exploration mission was carried out to survey a marine space with a surface and distance to the base station equivalent to the Cabo de Palos and Islas Hormigas Marine Reserve. The objective was to detect, track, and identify suspicious vessels within the area to validate the proposed architecture and the SAAO. 

**Figure 15:**
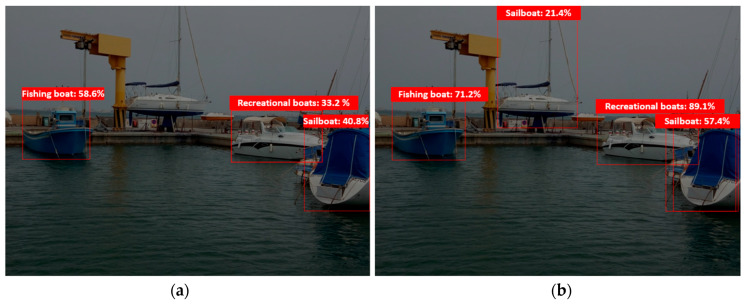
Edge (**a**) and cloud (**b**) trained model recognition tests.

The recognition system was tested in port before the mission to ensure that both Azure cloud and edge AI sources worked through the main bow camera as expected (Figure 15). A fishing boat, a recreational boat and a sailing boat were detected by the edge model, and an extra sailing boat by the cloud model, with better accuracy in all detections.

**Figure 16:**
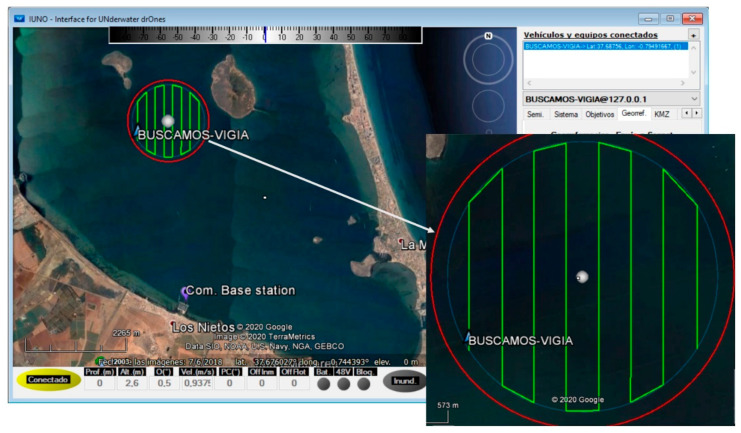
Start of mission (MMM) of surveillance of area equivalent to integral reserve.

The defined inspection area has a radius of 915 m, with a surface area of 2.63 km^2^ and a center at coordinates 37.689634° N and 0.787109° W. To avoid detecting vessels outside the test area due to the vision field, the area covered by the main mission was reduced by a radius of 100 m, as shown in Figure 16. The mission was planned on the IUNO platform. 

The ASV was remotely monitored and supervised from the fishing boat used as the auxiliary vessel for safety reasons during the entire mission. The auxiliary vessel was also used to verify the detection, recognition, and tracking capabilities implemented, as explained below. The different systems (control, lighting, thrusters, communications, vision, etc.) were tested before the BUSCASMOS-VIGIA mission. After successful validation, the mission was transferred from IUNO to the ASV and launched, and the vehicle headed for the starting point. Surveillance of the area began, following the previously defined route (Figure 16). From the first point of the mission to the fifth sweep in the Main Mission Mode (MMM) no incidents or detections occurred.

**Figure 17:**
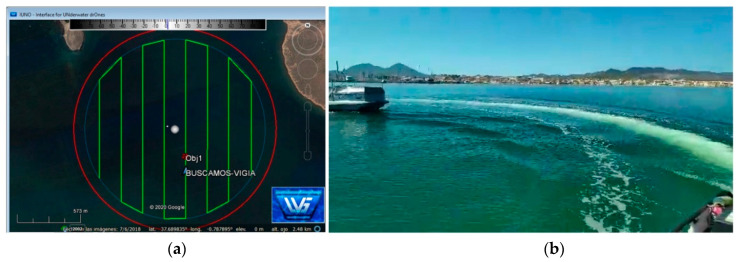
(**a**) Stopped vessel detected. Start *TM* mode. (**b**) Tracking Mode (*TM*) test during the experiment.

The technical team on board the fishing boat remained at a specific point in the fifth sweep to study the ASV’s behavior. The fishing boat was detected by BUSCAMOS-VIGIA (Figure 17a) and classified as a possible risk.

According to SAAO logs during this stage, all image analyses were performed by the cloud-trained model, except for one case in which the edge-trained model was used due to high latency. The target vessel’s behavior was studied to determine if it was stationary, according to the rules defined in Stage 2, the Fixed Buoy Mode (FBM). The registers showed that only the edge model was used. The bounding boxes of all the analyzed images were determined because the accuracy was high enough at this stage. After the FBM stage, when the results determined that the target vessel was stationary, the Tracking Mode (*TM*) stage began. The technical team on board the target vessel then started the motors to verify the tracking capabilities (Figure 17b).

The ASV successfully reached the target. As in FBM, only the edge model was used by the SAAO during the entire TM stage. The vehicle stopped over 15 m away and changed to Inspection Mode (*IM*), the last mission stage.

The results obtained from the Azure Cloud AI General Model on additional information about the target vessel were as follows: three people on board were detected. The automatically generated sentences “a group of people on a boat” and “a group of people riding on the back of a boat in the water” by the cloud general model were useful for obtaining details of the target vessel without the need to view cameras in real time and without human intervention. At this stage, the records show that the SAAO used both the cloud general model and the edge-trained model. The cloud results were not fast enough to determine the target vessel’s bounding box in some cases. Table 5 shows a summary of the SAAO logs during the experiment:

When the fishing boat left the area, BUSCAMOS-VIGIA ended the IM stage and continued with the planned mission (in MMM) until the eighth sweep was completed. The vessel was then commanded to return to port and no further incidents were registered during the rest of the mission.

## 6. Conclusions

This paper proposes an autonomous marine robot for protection and permanent surveillance in marine protected areas based on AI recognition. The robot was designed to survey and inspect marine reserves through AI-based image recognition services in search of vessels carrying out suspicious activities. Azure cloud computing and Azure edge computing services were used for image analysis, each of which has its own advantages and disadvantages, mainly related to accuracy and latency. At each stage of the marine surveillance mission, either accuracy or latency must be given priority. However, in marine scenarios it is difficult to ensure the stability of measurements and results, and certainty is far from being guaranteed.

Edge computing topology reduces latency in low-bandwidth environments. Cloud computing topology improves accuracy at the expense of increased latency. To meet the system’s requirements, we proposed and developed a smart algorithm to optimize autonomy by selecting the appropriate AI technology for the scenario being monitored. This proved to be crucial in deciding the best source of artificial intelligence to be used to achieve the specified objectives in each stage in real time. The smart algorithm (SAAO) ensures a trade-off between latency and accuracy.

As shown, accuracy results in low light conditions may affect the SAAO decisions. Solutions based on infrared auxiliary cameras and retraining of the proposed models for vessel recognition through infrared images would be a proper way to overcome these conditions without the need to modify the tracking algorithm or the proposed architecture. This will be the subject of further research.

## Figures and Tables

**Table 1:** Definition of mission stages.

	Stage 1 Main Mission Mode (MMM)	Stage 2 Fixed Buoy Mode (FBM)	Stage 3 Tracking Mode (TM)	Stage 4 Inspection Mode (IM)
**Priority**	Accuracy (recognition)	Latency	Latency	Both accuracy (recognition) and latency.
**Target**	Vessel recognition and classification	Bounding box study (size and positions) for a determined period. Remain in the same position with fixed heading.	Reach target vessel within defined limits.	Stay at a fixed distance and heading from target. Obtain general and additional information about target vessel.
**Output**	Is the target a risk vessel? (YES/NO)	Is the target vessel in the same position? (YES/NO) Alerts.	Has Target been reached inside defined limits? (YES/NO). Alerts. Save information.	Alerts. Obtain information of target vessel. Video streaming. Save information.

**Table 2:** Round trip delay (RTD) test of 300 samples of the edge and cloud models.

	Min Latency (s)	Max Latency (s)	Average (s)	Variance (s^2^)	Standard Deviation (s)
Cloud Model	0.805	5.298	1.412	0.872	0.934
Edge Model	0.213	0.896	0.336	0.012	0.108

**Table 3:** AI source preferences according to mission stage.

	Stage 1 Main Mission Mode (MMM)	Stage 2 Fixed Buoy Mode (FBM)	Stage 3 Tracking Mode (TM)	Stage 4 Inspection Mode (IM)
**Priority**	Accuracy (recognition)	Latency	Latency	Both accuracy (recognition) and latency.
**Preference**	Azure Cloud Custom Model	Azure Edge Custom Model	Azure Edge Custom Model	Azure Cloud General Model
**Alternative**	Azure Edge Custom Model	Azure Cloud Custom Model	Azure Cloud Custom Model	Azure Edge Custom Model

**Table 4:** Experimental SAAO results.

Image Samples	Edge		Cloud		SAAO Answer for Next Analysis			
	Lat. (s)	Acc. (%)	Lat. (s)	Acc. (%)	MMM	FBM	TM	IM
a	0.447	ND	1.412	16.7	CCM	CCM	CCM	CGM
b	0.418	64.3	1.814	68.7	CCM	ECM	ECM	CGM
c	0.324	19.5	2.816	50.5	ECM	ECM	ECM	CGM
d	0.356	47.9	1.313	65.8	CCM	ECM	ECM	CGM
e	0.403	23.1	4.211	33.8	ECM	ECM	ECM	ECM
f	0.392	36.8	1.284	55.7	CCM	ECM	ECM	ECM

ND—Not detected. CCM—cloud custom model. ECM—edge custom model. CGM—cloud general model.

**Table 5:** Summary of SAAO logs during the experiment.

Stage	Edge (Custom Model)				Cloud (Custom and General Model)				SAAO Answers
	Av. Lat. (s)	Av. Acc. (%)	No. uses	Detections	Av. Lat. (s)	Av. Acc. (%)	No. uses	Detections	Use of the Preferred AI Source (%)
MMM	0.220	21	1	1	1.521	34	2414	1	99.95
FBM	0.205	31	58	58	-	-	0	-	100.00
TM	0.231	39	436	436	-	-	0	-	100.00
IM	0.218	57	29	27	1.589	68	92	88	76.03

## Data Availability

The data presented in this study are available on request from the corresponding author.
